# RGB images-based vegetative index for phenotyping kenaf (*Hibiscus cannabinus* L.)

**DOI:** 10.1371/journal.pone.0256978

**Published:** 2021-09-07

**Authors:** Gyung Doeok Han, GyuJin Jang, Jaeyoung Kim, Dong-Wook Kim, Renato Rodrogues, Seong-Hoon Kim, Hak-Jin Kim, Yong Suk Chung

**Affiliations:** 1 Department of Plant Resources and Environment, Jeju National University, Jeju, Republic of Korea; 2 Department of Biosystems & Biomaterials Science and Engineering, College of Agriculture and Life Sciences, Seoul National University, Seoul, Republic of Korea; 3 Institute of Mathematics and Statistics, Federal University of Goias, Goiania, Brazil; 4 National Agrobiodiversity Center, National Institute of Agricultural Sciences, RDA, Jeonju, Republic of Korea; Shahjalal University of Science and Technology, BANGLADESH

## Abstract

Kenaf (*Hibiscus cannabinus* L.) is an industrial crop used as a raw material in various fields and is cultivated worldwide. Compared to high potential for its utilization, breeding sector is not vigorous partially due to laborous breeding procedure. Thus, efficient breeding methods are required for varieties that can adapt to various environments and obtain optimal production. For that, identifying kenaf’s characteristics is very important during the breeding process. Here, we investigated if RGB based vegetative index (VI) could be associated with traits for biomass. We used 20 varieties and germplasm of kenaf and RGB images taken with unmanned aerial vehicles (UAVs) for field selection in early and late growth stage. In addition, measuring the stem diameter and the number of nodes confirmed whether the vegetative index value obtained from the RGB image could infer the actual plant biomass. Based on the results, it was confirmed that the individual surface area and estimated plant height, which were identified from the RGB image, had positive correlations with the stem diameter and node number, which are actual growth indicators of the rate of growth further, biomass could also be estimated based on this. Moreover, it is suggested that VIs have a high correlation with actual growth indicators; thus, the biomass of kenaf could be predicted. Interstingly, those traits showing high correlation in the late stage had very low correlations in the early stage. To sum up, the results in the current study suggest a more efficient breeding method by reducing labor and resources required for breeding selection by the use of RGB image analysis obtained by UAV. This means that considerable high-quality research could be performed even with a tight budget. Furthermore, this method could be applied to crop management, which is done with other vegetative indices using a multispectral camera.

## Introduction

Kenaf *(Hibiscus cannabinus* L.) is an important industrial crop worldwide [1]. The total area in the world for kenaf, jute (*Corchorus olitorius*), and allied fiber were 1.50 mha, with a total annual production of 3.38 million tonnes in 2019 (http://www.fao.org/faostat/en/#data/QC). The crop is a fiber source for packaging, textiles, cushions, doors, fiberboards, absorption materials, insulation mats, carpet backings, animal bedding, and paper and pulp industries [2, 3]. In addition, kenaf has been shown to have great potential for the production of bio-energy [4]. Compared to other industrial fiber crops, kenaf has widespread adaptability in various soils and climates [5]. Kenaf is a fast-growing plant that can reach 4–6 m in height, with an average increase of 10 cm in a day [6].

Accurate plant phenotyping is essential for linking the environment and plant genetic characteristics to accelerate crop breeding [7–9]. However, high labor costs remit a single measurement of the final yield in diverse testing environments over multiple seasons in crop breeding programs [10]. Field monitoring using remote sensing technologies has been introduced to address this problem [11]. A black and white (B&W) rendition of an aerial color-infrared video composite was useful for detecting initial plant stress and for monitoring the progression of Phymatotrichum root rot and RKN/soil fungi complex damage [12]. Also, using airborne multi-spectral images produce various indices or images, which can be used to evaluate biomass, crop health, biotypes, and pest infestations in agricultural fields [13]. One such technology is the unmanned aerial vehicle (UAV) technology, which is rapidly developing and suggesting new methods for precise crop monitoring [14]. RGB (Red, green and blue) images obtained from cameras mounted on UAVs can be utilized for monitoring crop growth, development, and round coverage ratio [15–17]. Furthermore, visible remote sensing ramie (*Boehmeria nivea* L.) images from UAV showed that the germplasm resources had rich variation and wide diversity [18]. The various vegetation indices (VIs) can be derived from RGB images, which show good performance for evaluating various traits, including biomass [15].

In this study, (i) we investigated traits of randomly selected 20 kenaf entries to determine what traits affect biomass production. Those tratis include surface area, stem diameter, and the number of nodes manually. Furthermore, we estimated plant height from RGB images and widely used RGB-based VIs, such as the excess green index (EXG), excess green minus excess red index (EXGR), excess red index (EXR), normalized difference index (NDI), green leaf index (GLI), and the visible atmospheric resistant index (VARI) using UAVin order to determine if any traits abtained above are associated with biomass of kenaf in the early and late growth stage. If they are asscociated, it would be helpful for developing pre-breeding matetials for biomass in kenaf.

## Materials and methods

### Experiment site and plant materials

**Fig 1:**
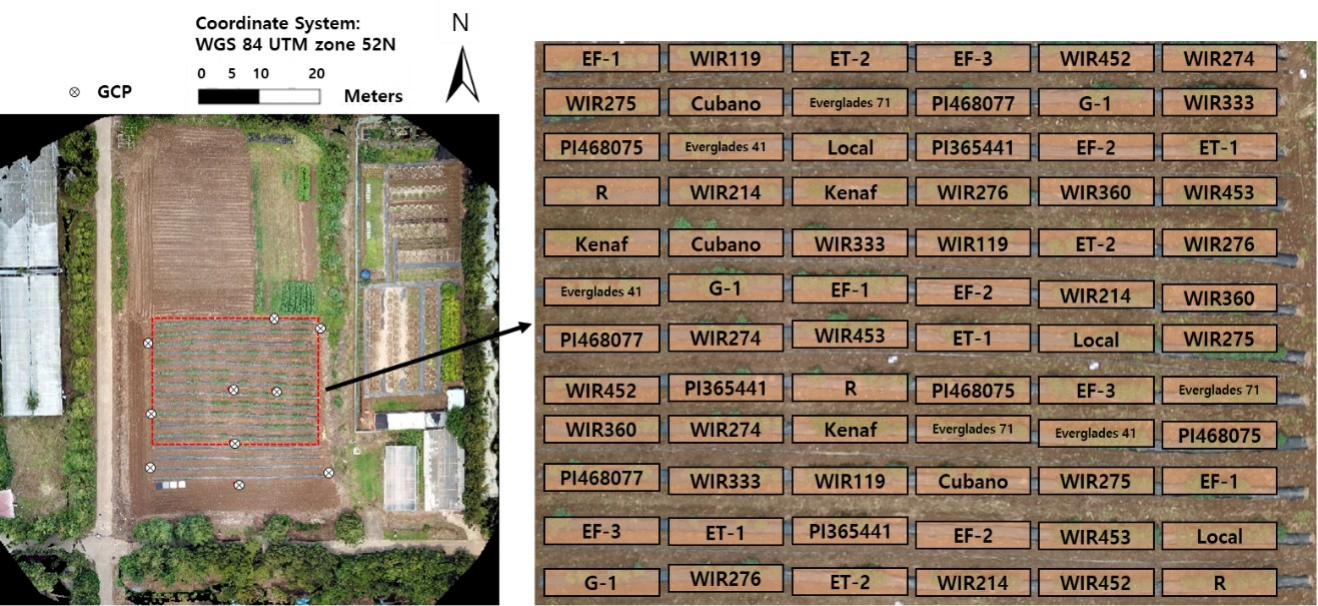
Experimental plots and total cultivars of kenaf used in this study.

The remote sensing study was conducted within a field in Jeju National University (33°72’N, 126°33’E, altitude 257m), located in 102, Jejudaehak-ro, Jeju-si, Jeju-do, Republic of Korea during the growing season in 2019 (June to August) (Fig 1). On May 5, 2019 twenty-four cultivars of kenaf were planted randomly with three replication in totally 72 plots. Each plot contained fifteen kenaf seeds 25 cm apart. Watering was started 14 days after planting, once a day, till the end of the experiment. Black plastic mulch films were used to reduce damage from weed in growing season. One data was extracted from each plot for phenotyping resulting to acquire three data form each genotype in every UAV flight. While germination was generally 80–90%, entry ‘EF-2’ did not germinate. In addition, lodging caused by typhoons was solved by using crutches.

For accurate geo-referencing of UAV images, a set of ground control points (GCPs) made with 21.0 by 29.7 cm paper sheets were distributed across the study field. The GCP locations were measured with a C099-F9P based on real-time kinematic-global system (RTK-GPS) providing positioning precision within 0.01 m horizontal and vertical precision.

### Data acquisition

The aerial images of the experimental field at each set were captured using the unmanned aerial vehicle (UAV) as the MAVIC 2 Pro (SZ DJI Technology Co., China), the UAV which mounted RGB sensors recording the radiation values in the red (~600 nm), green (~550 nm), and blue (~450 nm) spectral bands with 2D array pixels (L1D-20c, Hasselblad, Sweden). The RGB camera acquired 12-megapixels (4000×3000) using a 1/2.3ˮ CMOS sensor and a 24 mm-48 mm zoom lens. The angular field of views (FOVs) is 72.3° horizontal and 57.5° vertical, generating approximately 1.4 cm ground sampling distance (GSD) at 40 m above ground level. Before planting, an image of an empty field was taken for the estimation of the digital terrain model (DTM).

The UAV platform was set to automatically fly tracking waypoints and acquire aerial images over the experimental plot using Pix4Dcapture (Pix4D SA, Lausanne, Switzerland), free UAV flight planning application on mobile phone. In each flight, Pix4D software triggered image acquisition including position data based on global navigation satellite systems (GNSS). The flight missions were conducted to cover the whole experimental plot and acquire images with slow speed, 90% overlap and 40 m altitude set in Pix4Dcapture. However only on 22 June the flight altitude was 20m and low overlapping ratio (70%) was set in Pix4Dcapture (Table 1).

**Table 1:** UAV aerial images acquisition during growing seasons (June to August).

Image date	Flight Altitude (m)	The number of Images	Covering Area (*m*^2^)	Ground Sampling Distance (cm/pixel)	Illumination	Wind (m/s)
22062019 (50 DAS)	20	74	4300	0.69	Mist	3.9
14072019 (72 DAS)	40	77	9000	1.43	Mist	2.7
26072019 (85 DAS)	40	68	7800	1.39	Cloudy	3.9
06082019 (99 DAS)	40	97	10600	1.34	Cloudy	3.2
31082019 (121 DAS)	40	93	9100	1.40	Clear	2.7

Moreover, to compare and verify the UAV image data in the exact process, the number of nodes, main stem diameters, and plant height were measured using bare eyes, digital vernier calipers, and measuring tape immediately after the UAV flight. Three individuals of each entry were randomly selected from the plots. The number of nodes was measured by counting the number of nodes was measured by counting the points of the main stem where buds, leaves, and branching twigs originate, the stem diameter was estimated at the middle of the first and second nodes of the main stem, and the height was measured from the ground to the tip of the individuals using a measuring tape.

### Image processing

Images acquired on each date were processed in the semi-automated flow of the photogrammetry software Pix4Dmapper 3.0.17 (Pix4D SA, Lausanne, Switzerland) to generate orthoimage and digital surface model (DSM). Orthoimage and DSM were used for extraction of image variable related color and elevation information, respectively.

In the ‘Initial Processing’ step, key points between overlapping images were extracted based on geometrical similarities to aligning the images, estimating the camera position. After ‘Initial Processing’ step, the GCPs data based on RTK-GPS were imported to Pix4D program for geo-referencing of aerial images accurately. GCPs imported were located, and matched on aerial images manually, determining camera position for each image and correcting lens distortion. In the ‘Point Cloud and Mesh’ step, a dense 3D point cloud was generated based on 3D reconstruction among the key points extracted from aligned image set using Structure from Motion (SfM) algorithm. Then, 3D point cloud was interpolated to build the 3D mesh. In the ‘DSM, Orthomosaic and index’ step, 3D mesh was placed on real-world coordinate system, calculating elevation data for generating DSM. Then, orthoimage was generated through adding the color to 3D model by putting the aerial images on generated mesh. Finally, DSM and orthoimage were generated in the ‘GeoTIFF’ format including RGB pixel value and geographic information. The detail parameters in the processing are shown in Table 2.

**Table 2:** Parameters of the Pix4D processing used in this study.

Step	Items	Values
Initial Processing	Keypoits Image Scale	Full
	Matching Image Pairs	Aerial Grid of Corridor
	Targeted Number of Keypoits	Automatic
Point Cloud and Mesh	Point Cloud Image Scale	1
	Point Cloud Point Density	High
	Point Cloud Minimum Number of Matches	2
	3D Textured Mesh Settings	High Resolution
	Matching Window Size	7×7 pixels
DSM, Orthomosaic and index	DSM and Orthomosaic Resolution	Automatic
	DSM Filters	Use Noise Filtering Use Surface Smooting (Sharp)

**Fig 2:**
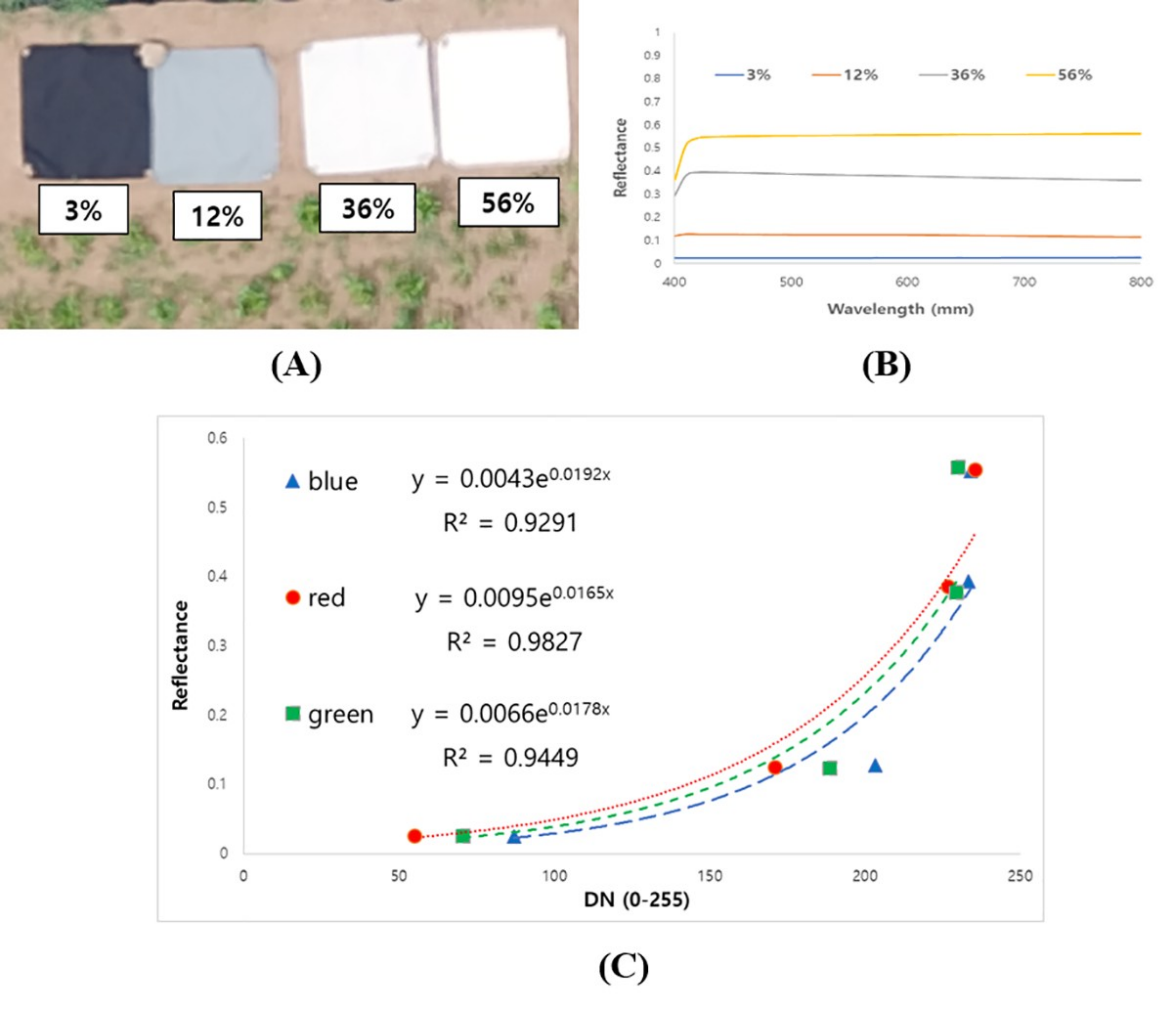
(A) Calibration targets within the experimental field; (B) Standard reflectance spectrum of the calibration targets; (C) Empirical Line Method (ELM) sample of the red, green and blue band mounted on the UAV (22 June 2019).

To reduce the effects of difference illumination and atmospheric conditions, a radiometric calibration of the aerial images was conducted using Empirical Line Method (ELM) on every flight. Calibration targets (1.2 by 1.2 m Group 8 Technology Type 822 ground calibration panels) with four scales (3%, 12%, 36%, and 56%) which provide standard reflectance values were placed in a location within the flight path to be captured by UAV platform (Fig 2). The target’s digital number (DN) values of each red, green and blue bands were extracted from orthoimage using ENVI software (L3 HARRIS Geospatial, USA). Then, the DNs of the calibration targets were fitted to the reflectance value, deriving Equation, exponential regression model:

$$r_{k}=A_{k}e^{B_{k}DN}$$ (1)

where *r_k_* represents the reflectance values of the acquired images, DN represents the digital number for each band, and *A_k_* and *B_k_* are the coefficients of the exponential regression model.

As a result, DNs in orthoimage on every flight were converted to standard reflectance value by applying Equation obtained at different dates. The coefficients of Equation on every flight date are introduced in Table 3.

**Table 3:** Results of the exponential regression model on each flight date.

Image date	Band	*A_k_*	*B_k_*	*R* ^2^
20190622 (50 DAS^a^)	R	0.0078	0.0181	0.98
	G	0.0104	0.0168	0.99
	B	0.0062	0.019	0.96
20190714 (72 DAS)	R	0.0095	0.0165	0.98
	G	0.0066	0.0178	0.94
	B	0.0043	0.0192	0.93
20190726 (85 DAS)	R	0.0078	0.0181	0.97
	G	0.0104	0.0168	0.99
	B	0.0062	0.019	0.96
20190806 (99 DAS)	R	0.0105	0.0158	0.99
	G	0.0087	0.0164	0.97
	B	0.0072	0.0174	0.97
20190831 (121 DAS)	R	0.0042	0.0211	0.96
	G	0.0043	0.021	0.94
	B	0.0037	0.0217	0.94

^a^ DAS represents day after seeding.

ENVI software (L3 HARRIS Geospatial, USA) was used to calculate plant height and vegetation indices. Plant height was estimated from the maximum values in each entry plot of the model that extracted the DTM from the digital surface models (DSMs) on July 14 and August 31 [15]. Six vegetation indices (VIs), excess green (EXG), excess green minus excess red (EXGR), excess red (EXR), green-red vegetation index (GLI), normalized difference index (NDI), and visible-band difference vegetation index (VARI), were calculated from the spectral band raster (Table 4). The region of interest (ROI) of each plot was identified, and the values of VIs were segmented by ROIs of each entry plot to estimate the mean pixel values of the indices per entry. The surface area of individual entry plots was produced from the plot surface areas divided by the number of plants in each plot, and the plot surface area was estimated using Otsu’s threshold for EXG [19].

**Table 4:** Formulas of vegetation indices.

VI	Formula^a^^,^ ^b^	Ref
EXG	2*g−r−b*	[20]
EXR	1.4*r−g*	[20]
NDI	(*g−r*)/(*g+r*)	[20]
EXGR	3*g*−2.4*r−b*	[20]
GLI	(2*g−b−r*)/(2*g+b+r*)	[21]
VARI	(*g−r*)/(*g+r−b*)	[21]

^a^ R = red band (630–690 nm), G = green band (520–600 nm), B = blue band (420–450 nm).

^b^ r = R/(R+G+B), g = G/(R+G+B), b = B/(R+G+B).

### Statistical analysis

All statistical analysis was performed by "R" software (Ver. 1.3.1056., RStudio Team, R Foundation for Statistical Computing, Boston). The data sets were checked for normality by the Shapiro-Wilk test. Because some data sets did not show a normal distribution, the non-parametric Kruskal-Wallis rank sum test followed by the Dunn-Bonferroni post hoc test was used to compare measured traits of 20 entries of kenaf. For the same reason, correlation analysis was carried out with the Spearman correlation test.

For a more comprehensive description of the results, principal component analysis (PCA) was used to summarize the relationship among the measure traits. In this experiment, the EXR value has a negative value. Since this indicator has the opposite meaning of the other VIs, PCA analysis was performed using the absolute value of EXR.

## Results

### Growth characteristic of 20 kenaf entries at two different stages

**Fig 3:**
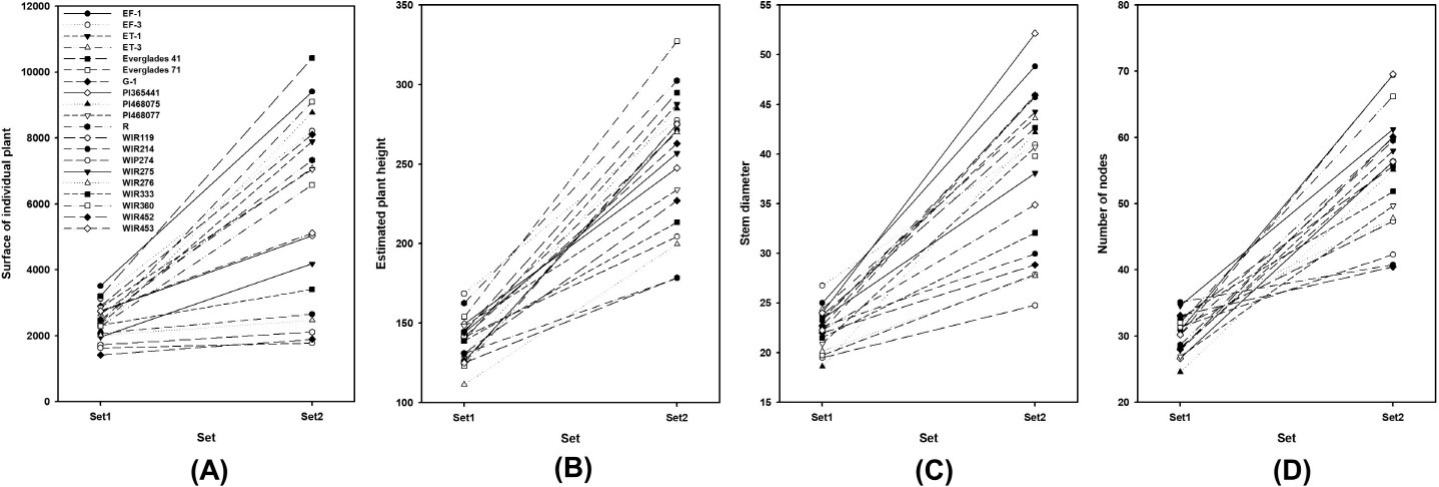
Growth of kenaf (*Hibiscus cannabinus* L.) entry traits affecting biomass production at two different growth stages. (A) Surface of the individual plant (cm^2^), (B) Expected plant height (mm), (C) Stem diameter (mm), and (D) Number of nodes of 20 kenaf entries at two different growth stages. Set 1 and Set 2 measured June 22 and August 31.

**Fig 4:**
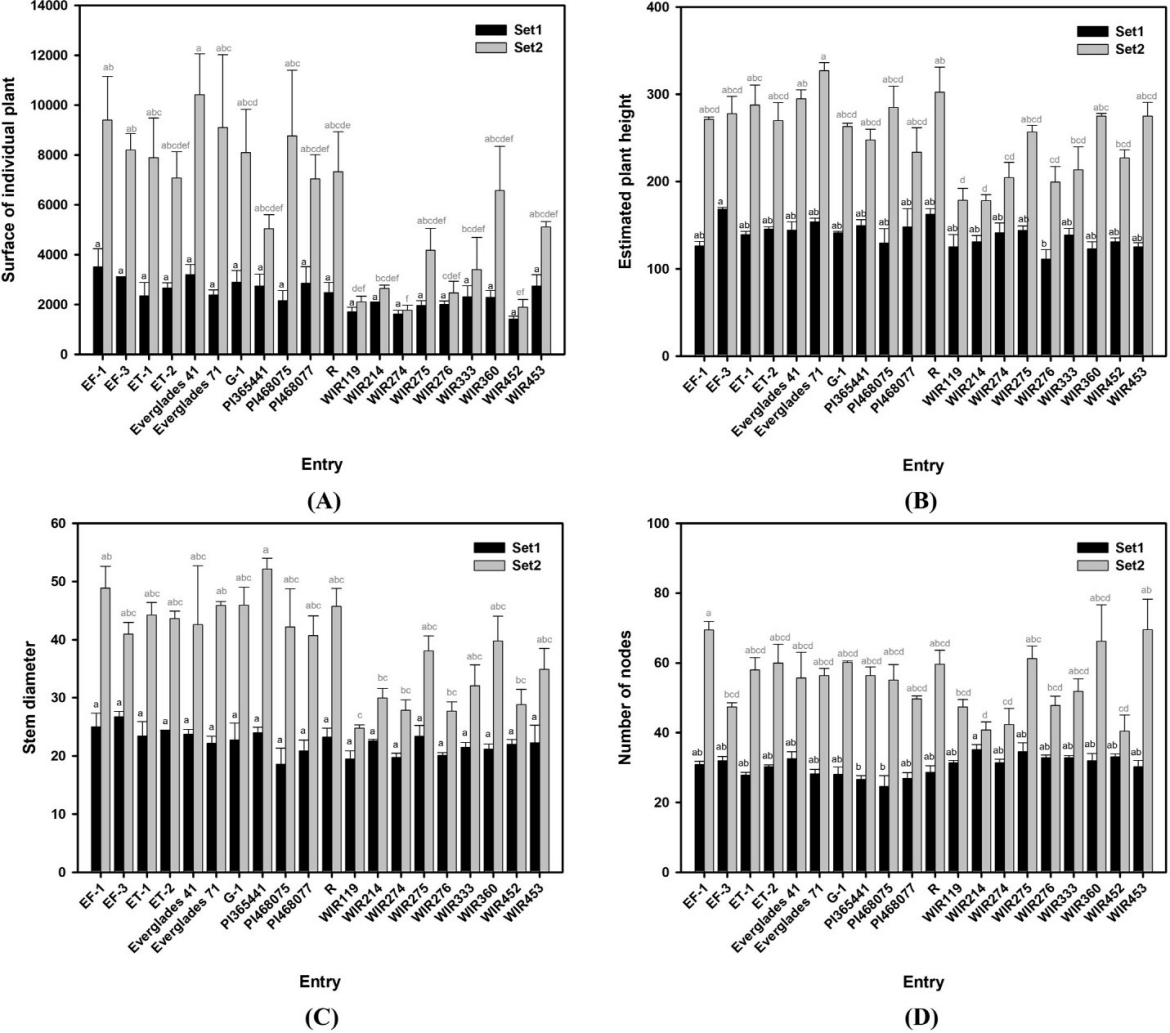
Variation of traits among kenaf (*Hibiscus cannabinus* L.) entry two growth stage. (A) Surface of the individual plant (cm^2^), (B) Expected plant height (mm), (C) Stem diameter (mm), and (D) Number of nodes of 20 kenaf entries at two different growth stages. Set 1 and Set 2 measured June 22 and August 31. The black bar and letter represent set 1, and the grey bar and letter represent set 2. Different letters on the bar are significantly different by Dunn test with Bonferroni adjustment. Non-parametric rank data were used for the statistical analysis; however, untransformed data are represented.

Table 5 shows that replications are not significantly different while the traits are significantly different between each entry except the surface area of individual plants in set 1 (*p* = 0.06). EF-1 which has the largest surface area in set 1 was approximately three times larger than that of the WIR425 which has the smallest surface area. However, in set 2, Everglades 41 which has the largest surface area was approximately five times larger than WIR274 which has the smallest surface area. There was also a change in ranking, but it could be seen that entries generally maintained a ranking between set 1 and set 2. Fig 4 suggests that, in particular, the entry "Everglade 71" ranks in the middle in set 1 but ranks 3rd out of the total 20 entries in set 2, suggesting that this entry grows faster than the others. The estimated plant height values fluctuated in rank between the set and the surface of individual plant areas but generally retained their rank (Figs 3 and 4). The rank of "Everglade 71" was maintained, but "WIR274" ranked in the middle in set 1 and 17th in set 2. For stem diameter, "Everglade 71" and "PI46875" showed rapid growth between sets (Figs 3 and 4). The most rapid rank fluctuation was observed among the traits observed in the number of nodes. The entry "WIR214" was the highest-ranked in set 1, but ranked 19th out of 20 entries in set 2. Similarly, entries "WIR452" and "WIR276" were highly ranked in set 1, but not in set 2. In contrast, entries "EF-1" and "G-1" showed low ranking in set 1 but ranked 2nd and 5th in set 2, respectively (Figs 3 and 4). The differences among the other entries increased between sets (Figs 3 and 4).

**Table 5:** Kruskal-Wallis rank sum test at two growth stages.

		Sur ^a^	Eph	Sd	Nn	EXG ^b^
Source	Df	Set1 ^c^	Set2	Set1	Set2	Set1
Rep	2	NS ^d^	NS	NS	NS	NS
Entry	19	NS	***	*	**	NS
		EXGR	EXR	GLI	NDI	VARI
Source	Df	Set1	Set2	Set1	Set2	Set1
Rep	2	NS	NS	NS	NS	NS
Entry	19	**	***	*	***	**

^a^ Sur = Surface area of individual plants (cm^2^); Eph = Estimated plant height (mm); Sd = Stem diameter (mm); and Nn = Number of nodes

^b^ EXG (excess green index), EXGR (excess green minus excess red index), EXR (excess red index), GLI (green leaf index), NDI (normalized difference index), and VARI (visible atmospheric resistant index).

^c^ Set 1 and Set 2 measured July 14 and August 31.

^d^ NS, nonsignificant at *p* ≤ 0.05

*significant at the 0.05

** significant at the 0.01

*** significant at 0.001.

### Growth indices of the 20 kenaf entries at two different stages

**Fig 5:**
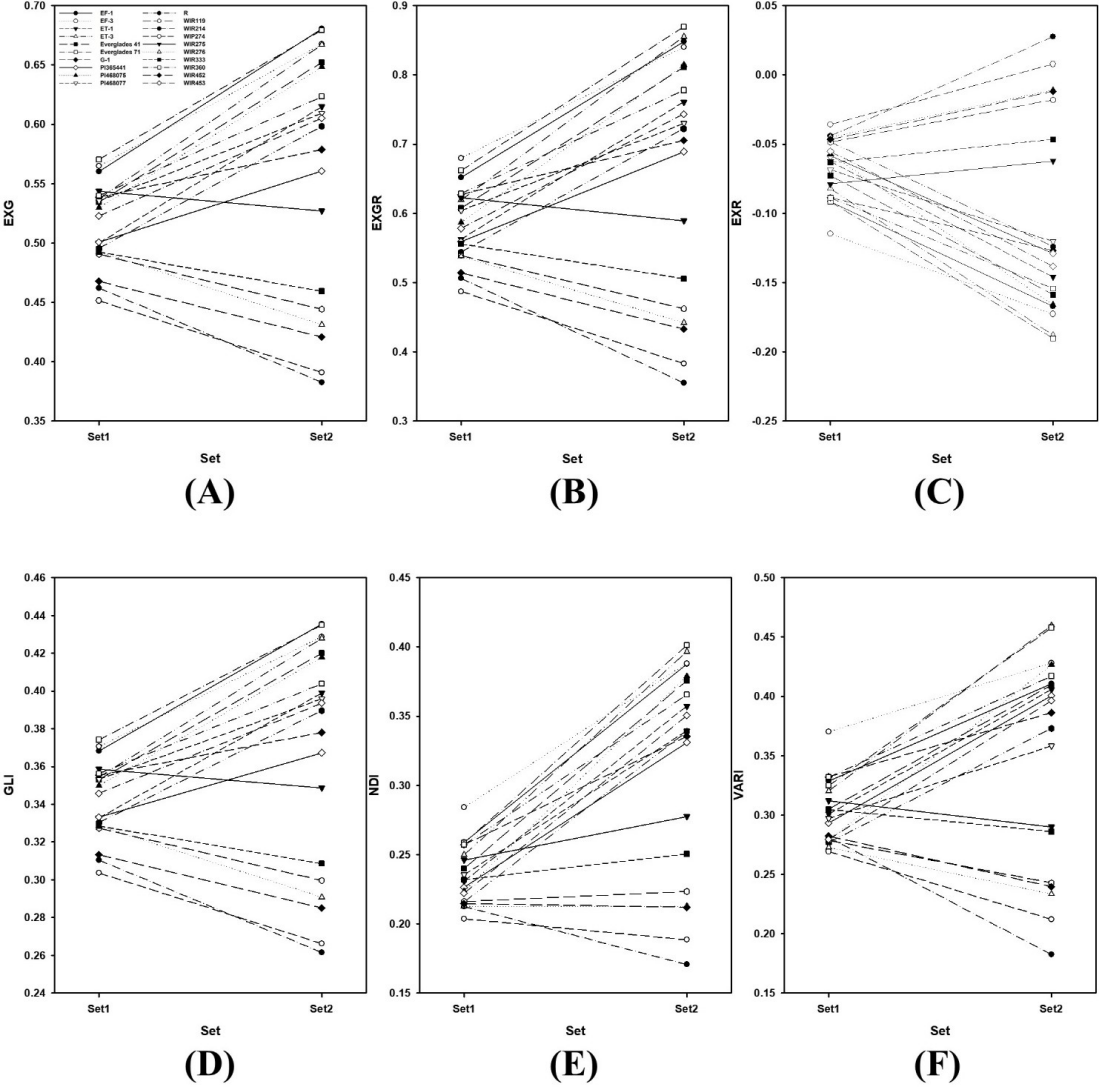
VIs of 20 kenaf entries at two different growth stages. (A) EXG, (B) EXGR, (C) EXR, (D) GLI, (E) NDI, and (F) VARI of 20 kenaf entries at two different growth stages. Set 1 and Set 2 measured June 22 and August 31.

**Fig 6:**
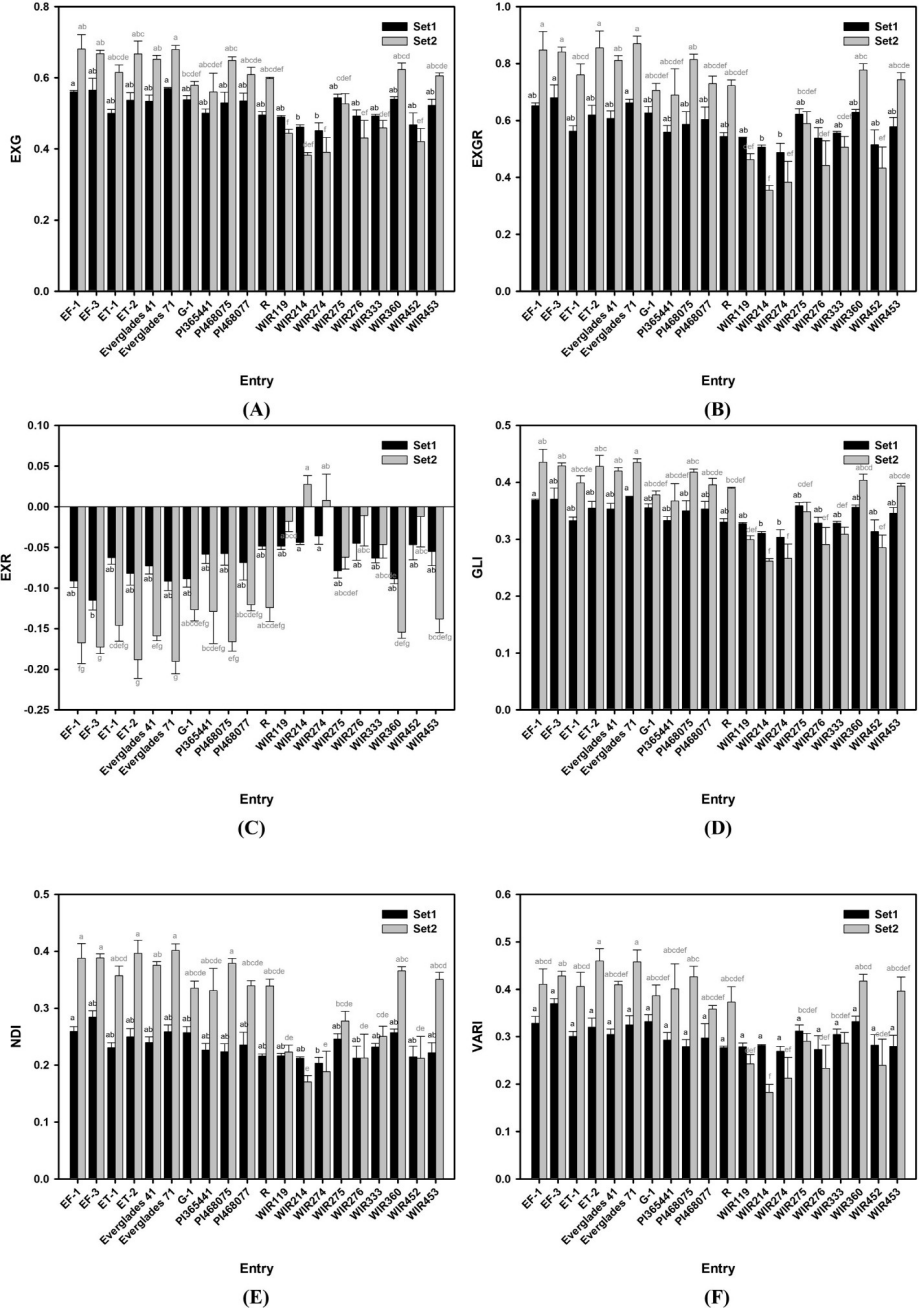
Variation of VIs among kenaf (*Hibiscus cannabinus* L.) entry two growth stage. (A) EXG, (B) EXGR, (C) EXR, (D) GLI, (E) NDI, and (F) VARI of 20 kenaf cultivars at two different growth stages. Set 1 and Set 2 measured June 22 and August 31. The black bar and letter represent set 1, and the grey bar and letter represent set 2. Different letters on the bar are significantly different by Dunn test with Bonferroni adjustment. Non-parametric rank data were used for the statistical analysis; however, untransformed data are represented.

Figs 5 and 6 shows the EXG, EXGR, EXR, GLI, NDI, and VARI changes between the sets. Except for entry in “C” presenting EXR values, all graphs in Fig 5 are similar. This is because the EXR value has the opposite meaning to that of the other VIs. In Fig 5, the rank of entries between sets fluctuates more than that between the VIs. The differences among other entries increased between sets (Figs 5 and 6).

### Spearman’s rank correlations among measured traits

Spearman’s rank correlations between an individual plant’s surface, estimated plant height, stem diameter, number of nodes, EXG, EXGR, EXR, GLI, NDI, and VARI are shown in Table 6. In set 1, the surface of the individual plant showed a moderate correlation with the stem diameter and VIs. However, this was not significant for the estimated plant height and number of nodes. In set 2, the surface of individual plants showed a high positive correlation (rho > 0.71) with all measured traits, except for the number of nodes that showed a moderate correlation (rho = 0.56) and EXR that showed negative correlation (rho = -0.73). The estimated plant height had a low correlation (rho < |0.28|) with measured traits, except for stem diameter, which showed a moderate correlation (rho = 0.54). Stem diameter had a low to moderate correlation in set 1 with the number of nodes and VIs. In set 1, Vis and the number of nodes were not correlated; however, in set 2, the number of nodes showed a moderate correlation with the VIs. Among the VIs, GLI and NDI showed a high correlation with the individual plant’s surface, estimated plant height, stem diameter, and node number. In addition, the correlation was high between VIs. In particular, EXR was highly negatively correlated with other VIs.

**Table 6:** Spearman’s rank correlation among surface of an individual plant, estimated plant height, actual plant height, stem diameter, number of nodes, EXG, EXGR, EXR, GLI, NDI, and VARI in kenaf germplasm at two growth stages.

		Eph	Sd	Nn	EXG	EXGR	EXR	GLI	NDI	VARI
Sur ^a^	Set1^b^	0.23 NS ^c^	0.66 ***	0.08 NS	0.50 ***	0.50 ***	-0.47 ***	0.50 ***	0.47 ***	0.43 ***
	Set2	0.73 ***	0.79 ***	0.56 ***	0.76 ***	0.76 ***	-0.73 ***	0.76 ***	0.75 ***	0.71 ***
Eph	Set1	1.00	0.54 ***	-0.07 NS	0.24 NS	0.27 *	-0.28 *	0.25 NS	0.27 *	0.25 NS
	Set2	1.00	0.61 ***	0.63 ***	0.76 ***	0.76 ***	-0.74 ***	0.76 ***	0.75 ***	0.73 ***
Sd	Set1	0.54 ***	1.00	0.32 *	0.37 **	0.41 **	-0.44 ***	0.38 **	0.44 ***	0.43 ***
	Set2	0.61 ***	1.00	0.69 ***	0.60 ***	0.61 ***	-0.61 ***	0.60 ***	0.61 ***	0.61 ***
Nn	Set1	-0.07 NS	0.32 *	1.00	-0.11 NS	-0.07 NS	0.01 NS	-0.11 NS	-0.01 NS	0.04 NS
	Set2	0.63 ***	0.69 ***	1.00	0.49 ***	0.50 ***	-0.52 ***	0.48 ***	0.51 ***	0.53 ***
EXG	Set1	0.24 NS	0.37 **	-0.11 NS	1.00	0.97 ***	-0.87 ***	1.00 ***	0.86 ***	0.77 ***
	Set2	0.76 ***	0.60 ***	0.49 ***	1.00	0.99 ***	-0.96 ***	1.00 ***	0.98 ***	0.89 ***
EXGR	Set1	0.27 *	0.41 **	-0.07 NS	0.97 ***	1.00	-0.96 ***	0.97 ***	0.95 ***	0.88 ***
	Set2	0.76 ***	0.61 ***	0.50 ***	0.99 ***	1.00	-0.99 ***	0.99 ***	1.00 ***	0.94 ***
EXR	Set1	-0.28 *	-0.44 ***	0.01 NS	-0.87 ***	-0.96 ***	1.00	-0.87 ***	-1.00 ***	-0.98 ***
	Set2	-0.74 ***	-0.61 ***	-0.52 ***	-0.96 ***	-0.99 ***	1.00	-0.96 ***	-0.99 ***	-0.98 ***
GLI	Set1	0.25 NS	0.38 **	-0.11 NS	1.00 ***	0.97 ***	-0.87 ***	1.00	0.86 ***	0.77 ***
	Set2	0.76 ***	0.60 ***	0.48 ***	1.00 ***	0.99 ***	-0.96 ***	1.00	0.98 ***	0.89 ***
NDI	Set1	0.27 *	0.44 ***	-0.01 NS	0.86 ***	0.95 ***	-1.00 ***	0.86 ***	1.00	0.98 ***
	Set2	0.75 ***	0.61 ***	0.51 ***	0.98 ***	1.00 ***	-0.99 ***	0.98 ***	1.00	0.96 ***

^a^ Surface of individual plant = Sur, estimated plant height = Eph, stem diameter = Sd, and number of nodes = Nn.

^b^ Set 1 and Set 2 measured June 14 and August 31.

^c^ NS, nonsignificant at *p* ≤ 0.05

*Significant at the 0.05

**Significant at the 0.01

*** Significant at the 0.001 probability level.

### PCA analysis of the measured traits

**Fig 7:**
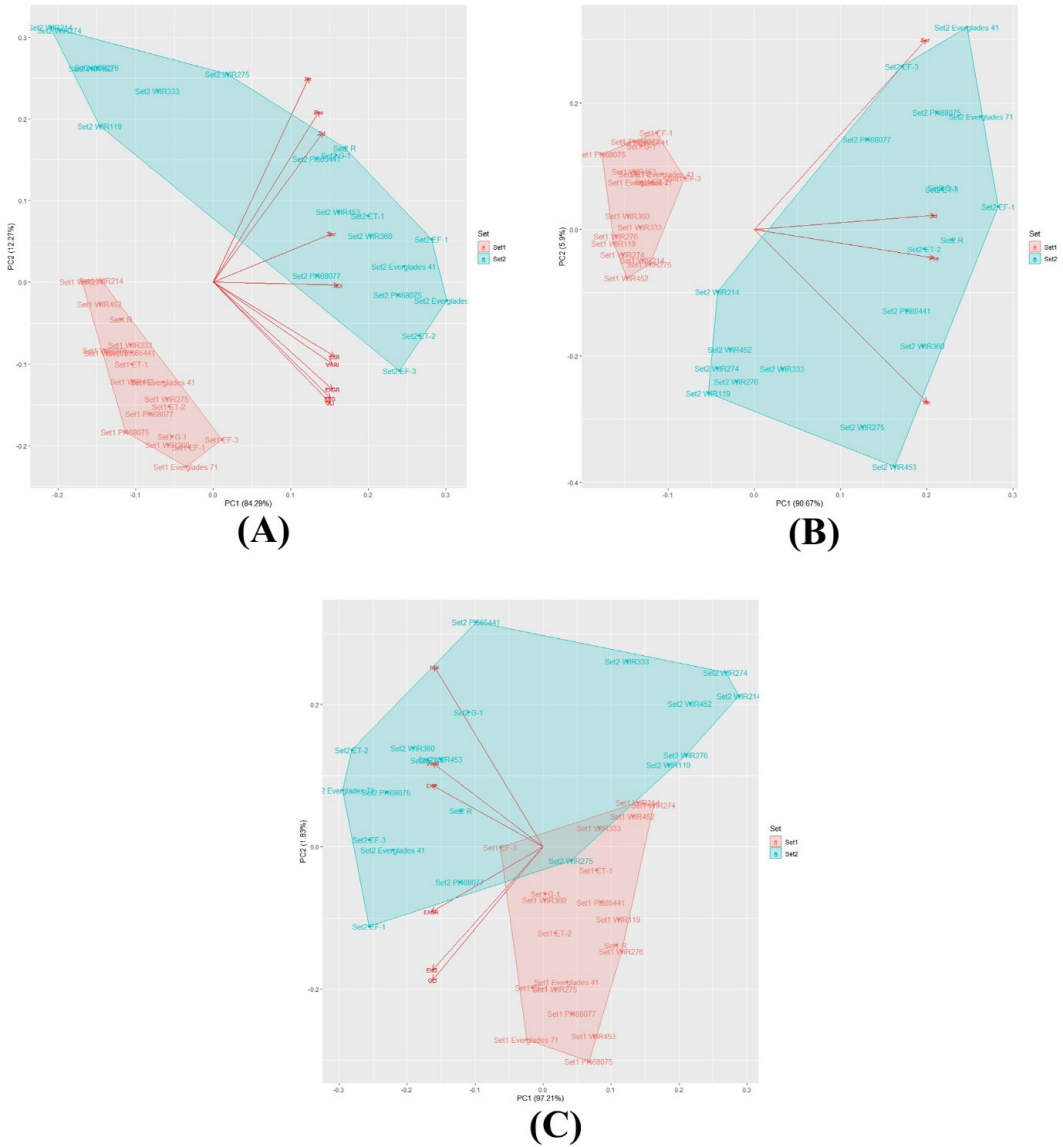
PCA analysis of 20 kenaf entries at two different growth stages. (A) PCA analysis by surface of individual plant, expected plant height, stem diameter, number of nodes, EXG, EXGR, EXR, GLI, NDI, and VARI; (B) PCA analysis by surface of individual plant, expected plant height, stem diameter, and number of nodes; (C) PCA analysis by EXG, EXGR, EXR, GLI, NDI, and VARI. Set 1, and set 2 measured June 22, and August 31.

The PCA indicated that the first two components accounted for 98.7% of growth traits with RGB-VIs, 96.56% of growth traits, and 99.24% of RGB-VIs (Table 7). In the PCA results of growth traits with RGB-VIs, all measured traits and RGB-Vis in the first component contributed similarly and accounted for 84.29% of the total variation. The second component was characterized by the estimated plant height and the number of nodes accounting for 12.27% of the variation (Fig 7A). One group was set 1, and the other was set 2. Most of the entries in set 1 are located in the left-down corner. In set 2, most of the entries except for WIP214, WIR274, WIR276, WIR453, WIR275, WIR333, and WIR119 are located on the right side. These entries are located on the right upper side, including the estimated plant height and number of nodes. In the PCA of growth traits, the first component accounted for 90.67% of the total variation. The second component was characterized by individual plant surfaces and the number of nodes accounting for 5.90% of the variation (Table 7). The scatter plot represents the entries in set 1 located left of the X-axis and those in set 2 located right of the X-axis and are more spread out than in set 1 (Fig 7B). The RGB-VIs PCA results show that the first components account for 97.21% of the total variation, with a similar rate of each VI. NDI, GLI, and EXG characterize the second component accounting for only 1.83% of the variation (Table 7). The PCA scatter plot shows that set 1 entries are located from -0.1 to 0.2 of the X-axis and are overlapped by set 2 entries which spread from -0.3 to 0.3 (Fig 7C).

**Table 7:** The eigenvalues of the correlation matrix for four Growth Traits (GT) and 5 RGB-based growth index (RGB-VI) on 20 entries of kenaf.

Traits	Principle components					
	GT+ RGB-VI		GT		RGB-VI	
	First (84.29%)	Second (12.27%)	First (90.67%)	Second (5.90%)	First (97.21%)	Second (1.83%)
Sur ^a^	0.327	0.127	0.486	0.733		
Eph	0.291	0.444	0.511	-0.110		
Sd	0.300	0.389	0.511	0.054		
Nn	0.262	0.532	0.491	-0.669		
EXG	0.322	-0.303			-0.409	-0.434
EXGR	0.327	-0.279			-0.413	-0.229
EXR	0.333	-0.194			-0.410	0.218
GLI	0.321	-0.314			-0.409	-0.470
NDI	0.343	-0.008			-0.401	0.635
VARI	0.328	-0.214			-0.407	0.296

^a^ Surface of individual plant = Sur, estimated plant height = Eph, stem diameter = Sd, and number of nodes = Nn.

## Discussion

This study aimed to confirm whether RGB images from UAVs could estimate biomass in kenaf planted in the field for breeding selection. If the image from the UAV successfully estimated kenaf biomass, the image could be used as a selection tool for kenaf breeding, saving considerable effort and costs for measuring the characteristics of field-planted kenaf.

We collected significant traits, such as the surface area of plants, height, stem diameter, node number, and RGB images to estimate each kenaf entry’s biomass (S1 Table). The surface area, plant height, and RGB-VI were calculated or estimated from the UAV image. In contrast, collecting stem diameter and node number data requires human effort, time, and resources. Similarly, in other crops, because of multiple environments over many seasons and replications, phenotyping of agricultural traits is recognized as the most laborious [22]. Interestingly, in kenaf, the growth traits had no or moderate correlation at the early stage (set 1); however, the stem diameter and node number had a significantly positive correlation with estimated plant height in set 2, that is, at the later stage (Table 6). Other studies have also reported that plant height, stem diameter, and node number are positively correlated with kenaf biomass [23, 24]. Therefore, the correlation between the plant surface area and height, which is advantageous for measurement, the kenaf biomass can be effectively predicted.

In addition, by using RGB-VIs, Kenaf biomass might be efficiently estimated. Except for EXR, VIs showed a strong positive correlation with the surface area of plants, estimated height, stem diameter, and node number. The chlorophyll in leaves and stems absorbs red light (centered around 0.67 μm) and blue light (centered around 0.45 μm), and reflects green light (centered around 0.55 μm), and the green digital image number in RGB images is larger than red or blue [25]. Since EXR is a VI, it emphasizes red and subtracts green; it negatively correlated with the measured kenaf traits. Furthermore, other VIs appeared to have similar positive correlations in set 2 with the surface area, plant height, stem diameter, and node number. In set 1, there was no or low correlation, suggesting that a late growth stage should be preferred when selecting plants for breeding based on VIs. Similar results were observed in the PCA. As shown in Fig 7, the entries in set 2 are scattered compared to set 1, which means that each entry’s characteristics appear better in the later stage.

Taken together, the results herein make it possible to estimate the biomass using kenaf’s surface area and estimated plant height using RGB images obtained from the UAV. In addition, it is possible to estimate biomass through VIs while minimizing the additional effort required for breeding selection. Hence, this study is unique in that it suggests a UAV operation with an RGB camera for breeding selection programs, especially those with a tight budget.

## Supporting information

S1 Fig Schematic drawing of ground and aerial measuring process. (TIF)

S1 Table Variation among kenaf (*Hibiscus cannabinus* L.) entry in the surface of individual plant, estimated plant height, stem diameter, number of nodes, EXG, EXGR, EXR, GLI, NDI and, VARI at two growth stages. (DOCX)
