# Correction: RGB images-based vegetative index for phenotyping kenaf (*Hibiscus cannabinus* L.)

**DOI:** 10.1371/journal.pone.0258567

**Published:** 2021-10-07

**Authors:** Gyung Doek Han, GyuJin Jang, Jaeyoung Kim, Dong-Wook Kim, Renato Rodrogues, Seong-Hoon Kim, Hak-Jin Kim, Yong Suk Chung

The first author’s name is spelled incorrectly. The correct name is: Gyung Doek Han.

In the Funding statement, the grant number from the funder Rural Development Administration, Republic of Korea is listed incorrectly. The correct grant number is: PJ01562003.

## References

[1] Han GD, Jang G, Kim J, Kim D-W, Rodrogues R, Kim S-H, et al. (2021) RGB images-based vegetative index for phenotyping kenaf (*Hibiscus cannabinus* L.). PLoS ONE 16(9): e0256978. 10.1371/journal.pone.0256978 34492059 PMC8423244

