# Supplementary figures and images for: RGB images-based vegetative index for phenotyping kenaf (Hibiscus cannabinus L.)
Source: PLoS One. 2021 Sep 7;16(9):e0256978. doi: 10.1371/journal.pone.0256978 (PMC8423244; doi:10.1371/journal.pone.0256978)

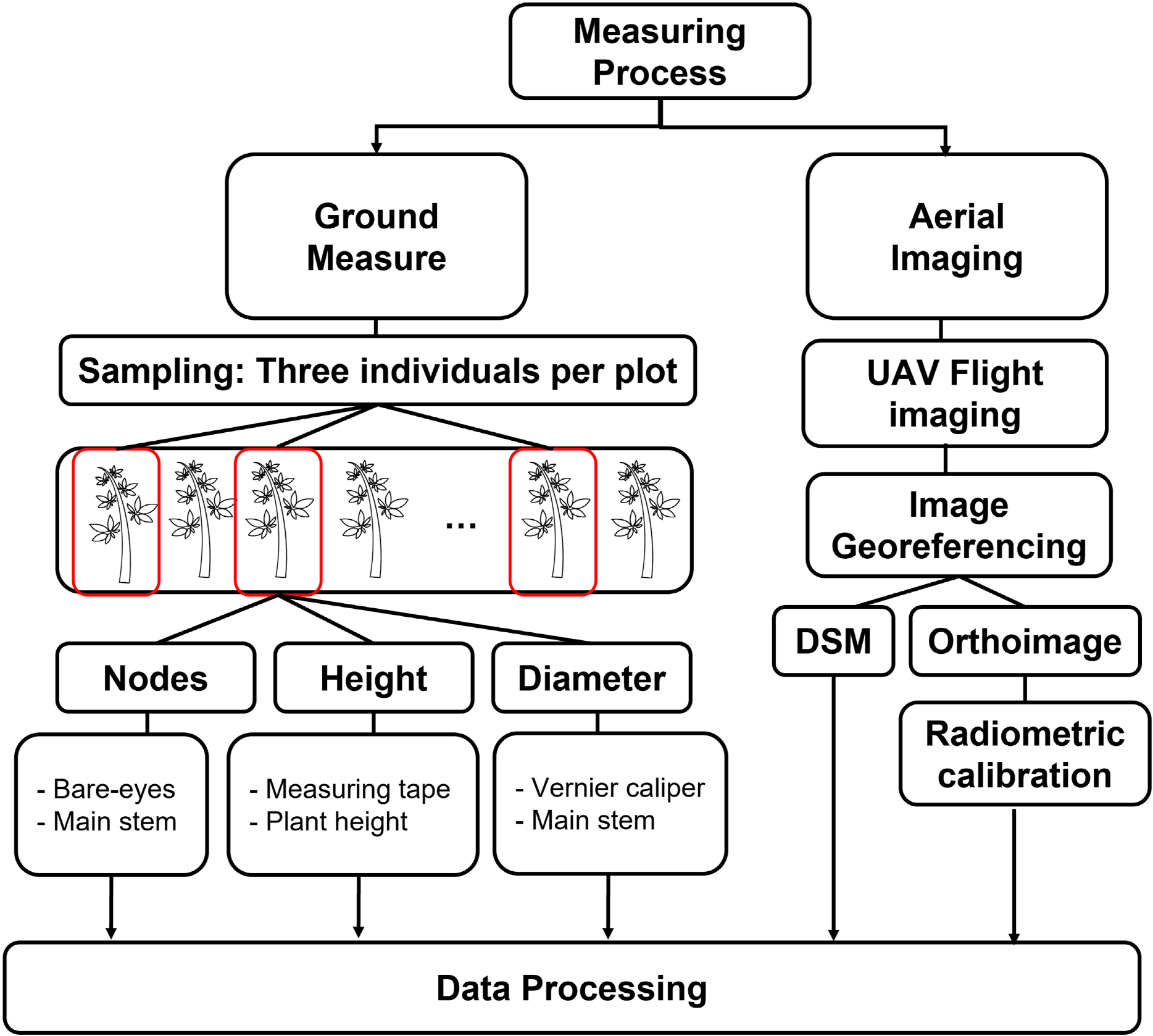

Supplement: S1 Fig — (TIF) [file pone.0256978.s001.tif]
