# Supplementary material for: RGB images-based vegetative index for phenotyping kenaf (Hibiscus cannabinus L.)
Source: PLoS One. 2021 Sep 7;16(9):e0256978. doi: 10.1371/journal.pone.0256978 (PMC8423244; doi:10.1371/journal.pone.0256978)
Supplement: S1 Table — (DOCX) [file pone.0256978.s002.docx]

**S1 Table. Variation among Kenaf (*Hibiscus cannabinus* L.) entry in the surface of individual plant, estimated plant height, stem diameter, number of nodes, EXG, EXGR, EXR, GLI, NDI and, VARI at two growth stages.**

| Surface of individual plant | | | | |  | Estimated plant height ^d^ | | | | |  | Stem diameter | | | | |  | Number of nodes | | | | |  | EXG | | | | |
| --- | --- | --- | --- | --- | --- | --- | --- | --- | --- | --- | --- | --- | --- | --- | --- | --- | --- | --- | --- | --- | --- | --- | --- | --- | --- | --- | --- | --- |
| Set1 ^a^ | |  | Set2 | |  | Set1 | |  | Set2 | |  | Set1 ^a^ | |  | Set2 | |  | Set1 | |  | Set2 | |  | Set1 | |  | Set2 | |
| Entry | Measured value |  | Entry | Measured value |  | Entry | Measured value |  | Entry | Measured value |  | Entry | Measured value |  | Entry | Measured value |  | Entry | Measured value |  | Entry | Measured value |  | Entry | Measured value |  | Entry | Measured value |
| EF-1 | 3,509.05 ± 736.82 a ^c^ |  | Ever 41 | 10,426.74 ± 1,638.48 a |  | EF-3 | 168.40 ± 1.87 a |  | Ever 71 | 327.24 ± 9.16 a |  | EF-3 | 26.74 ± 0.92 a ^c^ |  | PI365441 | 52.15 ± 1.85 a |  | WIR214 | 35.11 ± 1.44 a |  | WIR453 | 69.56 ± 8.66 ab |  | Ever 71 ^b^ | 0.57 ± 0.00 a ^c^ |  | EF-1 | 0.68 ± 0.04 ab |
| Ever 41 ^b^ | 3,198.75 ± 413.26 a |  | EF-1 | 9,405.10 ± 1,753.31 ab |  | R | 162.51 ± 6.59 ab |  | R | 302.52 ± 28.65 ab |  | EF-1 | 24.98 ± 2.36 a |  | EF-1 | 48.83 ± 3.77 ab |  | WIR275 | 34.56 ± 2.56 ab |  | EF-1 | 69.44 ± 2.44 a |  | EF-3 | 0.56 ± 0.03 ab |  | Ever 71 | 0.68 ± 0.01 a |
| EF-3 | 3,117.96 ± 2.66 a |  | Ever 71 | 9,100.40 ± 2,921.08 abc |  | Ever 71 | 153.84 ± 4.14 ab |  | Ever 41 | 294.97 ± 10.32 ab |  | ET-2 | 24.35 ± 0.06 a |  | G-1 | 45.94 ± 3.10 abc |  | WIR452 | 33.11 ± 0.87 ab |  | WIR360 | 66.22 ± 10.39 abcd |  | EF-1 | 0.56 ± 0.00 a |  | EF-3 | 0.67 ± 0.01 ab |
| G-1 | 2,900.05 ± 462.71 a |  | PI468075 | 8,767.25 ± 2,638.65 abc |  | PI365441 | 149.43 ± 7.02 ab |  | ET-1 | 287.74 ± 23.00 abc |  | PI365441 | 23.99 ± 0.95 a |  | Ever 71 | 45.86 ± 0.69 ab |  | WIR276 | 32.78 ± 0.80 ab |  | WIR275 | 61.22 ± 3.58 abc |  | WIR275 | 0.54 ± 0.01 ab |  | ET-2 | 0.67 ± 0.04 abc |
| PI468077 | 2,857.73 ± 669.41 a |  | EF-3 | 8,206.77 ± 655.34 ab |  | PI468077 | 148.02 ± 20.89 ab |  | PI468075 | 285.06 ± 24.10 abcd |  | Ever 41 ^b^ | 23.75 ± 0.82 a |  | R | 45.75 ± 3.04 abc |  | WIR333 | 32.78 ± 0.68 ab |  | G-1 | 60.11 ± 0.48 abcd |  | WIR360 | 0.54 ± 0.01 ab |  | Ever 41 | 0.65 ± 0.01 ab |
| WIR453 | 2,747.35 ± 453.78 a |  | G-1 | 8,103.46 ± 1,726.28 abcd |  | ET-2 | 145.40 ± 2.77 ab |  | EF-3 | 277.72 ± 20.06 abcd |  | ET-1 | 23.43 ± 2.47 a |  | ET-1 | 44.26 ± 2.14 abc |  | Ever 41 | 32.56 ± 2.00 ab |  | ET-2 | 60.00 ± 5.35 abcd |  | G-1 | 0.54 ± 0.01 ab |  | PI468075 | 0.65 ± 0.01 abc |
| PI365441 | 2,740.73 ± 483.78 a |  | ET-1 | 7,889.14 ± 1,594.56 abc |  | Ever 41 | 144.29 ± 9.71 ab |  | WIR453 | 275.24 ± 15.49 abcd |  | WIR275 | 23.39 ± 1.88 a |  | ET-2 | 43.60 ± 1.30 abc |  | EF-3 | 32.00 ± 1.17 ab |  | R | 59.56 ± 4.12 abcd |  | ET-2 | 0.54 ± 0.02 ab |  | WIR360 | 0.62 ± 0.02 abcd |
| ET-2 | 2,655.44 ± 226.48 a |  | R | 7,329.21 ± 1,611.26 abcde |  | WIR275 | 143.88 ± 5.35 ab |  | WIR360 | 275.22 ± 2.79 abc |  | R | 23.24 ± 1.56 a |  | Ever 41 | 42.61 ± 10.11 abc |  | WIR360 | 32.00 ± 2.04 ab |  | ET-1 | 58.00 ± 3.47 abcd |  | PI468077 | 0.54 ± 0.02 ab |  | ET-1 | 0.61 ± 0.02 abcde |
| R | 2,483.73 ± 412.24 a |  | ET-2 | 7,084.75 ± 1,045.36 abcdef |  | WIR274 | 141.34 ± 11.15 ab |  | EF-1 | 271.33 ± 2.82 abcd |  | G-1 | 22.70 ± 2.93 a |  | PI468075 | 42.16 ± 6.59 abc |  | WIR119 | 31.33 ± 0.69 ab |  | Ever 71 | 56.33 ± 2.04 abcd |  | Ever 41 | 0.53 ± 0.02 ab |  | PI468077 | 0.61 ± 0.02 abcde |
| Ever 71 | 2,381.06 ± 210.86 a |  | PI468077 | 7,043.54 ± 969.98 abcdef |  | G-1 | 141.16 ± 1.92 ab |  | ET-2 | 269.98 ± 20.52 abcd |  | WIR214 | 22.60 ± 0.28 a |  | EF-3 | 40.95 ± 2.01 abc |  | WIR274 | 31.33 ± 1.02 ab |  | PI365441 | 56.33 ± 2.50 abcd |  | PI468075 | 0.53 ± 0.03 ab |  | WIR453 | 0.61 ± 0.01 abcde |
| ET-1 | 2,353.28 ± 536.87 a |  | WIR360 | 6,575.11 ± 1,773.24 abcdef |  | ET-1 | 139.30 ± 3.53 ab |  | G-1 | 263.04 ± 3.73 abcd |  | WIR453 | 22.26 ± 3.01 a |  | PI468077 | 40.68 ± 3.41 abc |  | EF-1 | 30.89 ± 0.91 ab |  | Ever 41 | 55.67 ± 7.37 abcd |  | WIR453 | 0.52 ± 0.02 ab |  | R | 0.60 ± 0.00 abcdef |
| WIR333 | 2,312.42 ± 437.84 a |  | WIR453 | 5,116.53 ± 215.46 abcdef |  | WIR333 | 138.78 ± 7.49 ab |  | WIR275 | 256.80 ± 7.58 abcd |  | Ever 71 | 22.18 ± 1.21 a |  | WIR360 | 39.77 ± 4.29 abc |  | ET-2 | 30.22 ± 0.56 ab |  | PI468075 | 55.11 ± 4.41 abcd |  | PI365441 | 0.50 ± 0.01 ab |  | G-1 | 0.58 ± 0.01 bcdef |
| WIR360 | 2,286.03 ± 276.47 a |  | PI365441 | 5,041.12 ± 570.71 abcdef |  | WIR452 | 131.00 ± 4.52 ab |  | PI365441 | 247.65 ± 12.45 abcd |  | WIR452 | 21.98 ± 0.86 a |  | WIR275 | 38.07 ± 2.57 abc |  | WIR453 | 30.22 ± 1.82 ab |  | WIR333 | 51.83 ± 3.59 abcd |  | ET-1 | 0.50 ± 0.01 ab |  | PI365441 | 0.56 ± 0.05 abcdef |
| PI468075 | 2,154.33 ± 414.87 a |  | WIR275 | 4,182.02 ± 871.36 abcdef |  | WIR214 | 130.89 ± 7.29 ab |  | PI468077 | 233.81 ± 27.99 abcd |  | WIR333 | 21.50 ± 0.83 a |  | WIR453 | 34.91 ± 3.59 abc |  | R | 28.67 ± 1.84 ab |  | PI468077 | 49.67 ± 0.84 abcd |  | R | 0.50 ± 0.01 ab |  | WIR275 | 0.53 ± 0.03 cdef |
| WIR214 | 2,070.63 ± 47.35 a |  | WIR333 | 3,403.47 ± 1,287.52 bcdef |  | PI468075 | 129.67 ± 16.32 ab |  | WIR452 | 226.95 ± 9.27 bcd |  | WIR360 | 21.18 ± 0.87 a |  | WIR333 | 32.05 ± 3.63 abc |  | Ever 71 | 28.22 ± 1.28 ab |  | WIR276 | 47.78 ± 2.70 bcd |  | WIR276 | 0.49 ± 0.02 ab |  | WIR333 | 0.46 ± 0.02 def |
| WIR276 | 2,009.92 ± 140.22 a |  | WIR214 | 2,651.34 ± 122.65 bcdef |  | EF-1 | 126.20 ± 5.18 ab |  | WIR333 | 213.43 ± 26.56 bcd |  | PI468077 | 20.89 ± 1.82 a |  | WIR214 | 29.95 ± 1.65 bc |  | G-1 | 28.00 ± 2.14 ab |  | EF-3 | 47.42 ± 1.11 bcd |  | WIR333 | 0.49 ± 0.00 ab |  | WIR119 | 0.44 ± 0.01 def |
| WIR275 | 1,961.72 ± 202.46 a |  | WIR276 | 2,468.08 ± 467.55 cdef |  | WIR453 | 125.07 ± 4.82 ab |  | WIR274 | 204.43 ± 17.53 cd |  | WIR276 | 20.12 ± 0.43 a |  | WIR452 | 28.84 ± 2.60 bc |  | ET-1 | 27.89 ± 0.78 ab |  | WIR119 | 47.33 ± 2.22 bcd |  | WIR119 | 0.49 ± 0.00 ab |  | WIR276 | 0.43 ± 0.05 ef |
| WIR119 | 1,721.70 ± 175.99 a |  | WIR119 | 2,109.90 ± 228.39 def |  | WIR119 | 125.05 ± 14.19 ab |  | WIR276 | 199.58 ± 17.59 cd |  | WIR274 | 19.72 ± 0.74 a |  | WIR274 | 27.87 ± 1.79 bc |  | PI468077 | 26.89 ± 1.68 ab |  | WIR274 | 42.33 ± 4.58 cd |  | WIR452 | 0.47 ± 0.03 ab |  | WIR452 | 0.42 ± 0.04 ef |
| WIR274 | 1,621.79 ± 156.48 a |  | WIR452 | 1,889.37 ± 314.56 ef |  | WIR360 | 122.84 ± 7.90 ab |  | WIR119 | 178.50 ± 13.68 d |  | WIR119 | 19.49 ± 1.40 a |  | WIR276 | 27.72 ± 1.58 bc |  | PI365441 | 26.61 ± 1.11 b |  | WIR214 | 40.78 ± 2.31 d |  | WIR214 | 0.46 ± 0.01 b |  | WIR274 | 0.39 ± 0.04 f |
| WIR452 | 1,414.41 ± 120.38 a |  | WIR274 | 1,772.67 ± 207.97 f |  | WIR276 | 111.28 ± 10.83 b |  | WIR214 | 178.20 ± 6.83 d |  | PI468075 | 18.59 ± 2.73 a |  | WIR119 | 24.76 ± 0.56 c |  | PI468075 | 24.56 ± 3.09 b |  | WIR452 | 40.44 ± 4.58 cd |  | WIR274 | 0.45 ± 0.02 b |  | WIR214 | 0.38 ± 0.01 f |
| EXGR | | | | |  | EXR | | | | |  | GLI | | | | |  | NDI | | | | |  | VARI | | | | |
| Set1 ^a^ | |  | Set2 | |  | Set1 | |  | Set2 | |  | Set1 ^a^ | |  | Set2 | |  | Set1 | |  | Set2 | |  | Set1 | |  | Set2 | |
| Entry | Measured value |  | Entry | Measured value |  | Entry | Measured value |  | Entry | Measured value |  | Entry | Measured value |  | Entry | Measured value |  | Entry | Measured value |  | Entry | Measured value |  | Entry | Measured value |  | Entry | Measured value |
| EF-3 | 0.68 ± 0.05 a |  | Ever 71 | 0.87 ± 0.03 a |  | WIR274 | -0.04 ± 0.01 a ^c^ |  | WIR214 | 0.03 ± 0.01 a |  | Ever 71 | 0.37 ± 0.00 a |  | EF-1 | 0.44 ± 0.02 ab |  | EF-3 | 0.28 ± 0.01 a ^c^ |  | Ever 71 | 0.40 ± 0.01 a |  | EF-3 | 0.37 ± 0.01 a |  | ET-2 | 0.46 ± 0.03 a |
| Ever 71 | 0.66 ± 0.01 a |  | ET-2 | 0.86 ± 0.06 a |  | WIR214 | -0.04 ± 0.00 a |  | WIR274 | 0.01 ± 0.03 ab |  | EF-3 | 0.37 ± 0.02 ab |  | Ever 71 | 0.43 ± 0.01 a |  | EF-1 | 0.26 ± 0.01 ab |  | ET-2 | 0.40 ± 0.02 a |  | G-1 | 0.33 ± 0.01 a |  | Ever 71 | 0.46 ± 0.02 a |
| EF-1 | 0.65 ± 0.01 ab |  | EF-1 | 0.85 ± 0.06 a |  | WIR276 | -0.05 ± 0.02 ab |  | WIR276 | -0.01 ± 0.04 abc |  | EF-1 | 0.37 ± 0.00 a |  | EF-3 | 0.43 ± 0.01 ab |  | Ever 71 ^b^ | 0.26 ± 0.01 ab |  | EF-3 | 0.39 ± 0.01 a |  | WIR360 | 0.33 ± 0.01 a |  | EF-3 | 0.43 ± 0.01 ab |
| WIR360 | 0.63 ± 0.01 ab |  | EF-3 | 0.84 ± 0.02 a |  | WIR452 | -0.05 ± 0.02 ab |  | WIR452 | -0.01 ± 0.04 abc |  | WIR275 | 0.36 ± 0.01 ab |  | ET-2 | 0.43 ± 0.02 abc |  | G-1 | 0.26 ± 0.01 ab |  | EF-1 | 0.39 ± 0.03 a |  | EF-1 | 0.33 ± 0.01 a |  | PI468075 | 0.43 ± 0.02 abc |
| G-1 | 0.63 ± 0.02 ab |  | PI468075 | 0.81 ± 0.02 ab |  | R | -0.05 ± 0.00 ab |  | WIR119 | -0.02 ± 0.01 abcd |  | WIR360 | 0.36 ± 0.00 ab |  | Ever 41 | 0.42 ± 0.01 ab |  | WIR360 | 0.26 ± 0.01 ab |  | PI468075 | 0.38 ± 0.01 a |  | Ever 71 | 0.32 ± 0.02 a |  | WIR360 | 0.42 ± 0.01 abcd |
| WIR275 | 0.62 ± 0.02 ab |  | Ever 41 | 0.81 ± 0.02 ab |  | WIR119 | -0.05 ± 0.00 ab |  | WIR333 | -0.05 ± 0.02 abcde |  | G-1 | 0.36 ± 0.01 ab |  | PI468075 | 0.42 ± 0.01 abc |  | ET-2 | 0.25 ± 0.01 ab |  | Ever 41 | 0.38 ± 0.01 ab |  | ET-2 | 0.32 ± 0.02 a |  | EF-1 | 0.41 ± 0.03 abcd |
| ET-2 | 0.62 ± 0.03 ab |  | WIR360 | 0.78 ± 0.02 abc |  | WIR453 | -0.06 ± 0.02 ab |  | WIR275 | -0.06 ± 0.01 abcdef |  | ET-2 | 0.35 ± 0.01 ab |  | WIR360 | 0.40 ± 0.01 abcd |  | WIR275 | 0.25 ± 0.01 ab |  | WIR360 | 0.37 ± 0.01 abc |  | WIR275 | 0.31 ± 0.01 a |  | Ever 41 | 0.41 ± 0.01 abcdef |
| Ever 41 | 0.61 ± 0.03 ab |  | ET-1 | 0.76 ± 0.04 abcd |  | PI468075 | -0.06 ± 0.01 ab |  | PI468077 | -0.12 ± 0.01 abcdefg |  | PI468077 | 0.35 ± 0.01 ab |  | ET-1 | 0.40 ± 0.01 abcde |  | Ever 41 | 0.24 ± 0.01 ab |  | ET-1 | 0.36 ± 0.02 abcd |  | Ever 41 | 0.30 ± 0.01 a |  | ET-1 | 0.41 ± 0.03 abcde |
| PI468077 | 0.60 ± 0.04 ab |  | WIR453 | 0.74 ± 0.02 abcde |  | PI365441 | -0.06 ± 0.01 ab |  | R | -0.12 ± 0.02 abcdefg |  | Ever 41 | 0.35 ± 0.01 ab |  | PI468077 | 0.40 ± 0.01 abcde |  | PI468077 | 0.24 ± 0.02 ab |  | WIR453 | 0.35 ± 0.01 abcd |  | WIR333 | 0.30 ± 0.01 a |  | PI365441 | 0.40 ± 0.05 abcdef |
| PI468075 | 0.59 ± 0.04 ab |  | PI468077 | 0.73 ± 0.03 abcdef |  | ET-1 | -0.06 ± 0.01 ab |  | G-1 | -0.13 ± 0.01 abcdefg |  | PI468075 | 0.35 ± 0.02 ab |  | WIR453 | 0.39 ± 0.00 abcde |  | WIR333 | 0.23 ± 0.01 ab |  | PI468077 | 0.34 ± 0.01 abcde |  | ET-1 | 0.30 ± 0.01 a |  | WIR453 | 0.40 ± 0.03 abcdef |
| WIR453 | 0.58 ± 0.03 ab |  | R | 0.72 ± 0.02 abcdef |  | WIR333 | -0.06 ± 0.01 ab |  | PI365441 | -0.13 ± 0.04 bcdefg |  | WIR453 | 0.35 ± 0.01 ab |  | R | 0.39 ± 0.00 abcdef |  | ET-1 | 0.23 ± 0.01 ab |  | R | 0.34 ± 0.01 abcde |  | PI468077 | 0.30 ± 0.03 a |  | G-1 | 0.39 ± 0.02 abcdef |
| ET-1 | 0.56 ± 0.02 ab |  | G-1 | 0.71 ± 0.03 abcdef |  | PI468077 | -0.07 ± 0.02 ab |  | WIR453 | -0.14 ± 0.02 bcdefg |  | PI365441 | 0.33 ± 0.01 ab |  | G-1 | 0.38 ± 0.01 bcdef |  | PI365441 | 0.23 ± 0.01 ab |  | G-1 | 0.34 ± 0.01 abcde |  | PI365441 | 0.29 ± 0.02 a |  | R | 0.37 ± 0.03 abcdef |
| PI365441 | 0.56 ± 0.02 ab |  | PI365441 | 0.69 ± 0.09 abcdef |  | Ever 41 ^b^ | -0.07 ± 0.01 ab |  | ET-1 | -0.15 ± 0.02 cdefg |  | ET-1 | 0.33 ± 0.01 ab |  | PI365441 | 0.37 ± 0.03 abcdef |  | PI468075 | 0.22 ± 0.01 ab |  | PI365441 | 0.33 ± 0.04 abcde |  | WIR452 | 0.28 ± 0.02 a |  | PI468077 | 0.36 ± 0.01 abcdef |
| WIR333 | 0.56 ± 0.01 ab |  | WIR275 | 0.59 ± 0.04 bcdef |  | WIR275 | -0.08 ± 0.01 ab |  | WIR360 | -0.15 ± 0.01 defg |  | R | 0.33 ± 0.01 ab |  | WIR275 | 0.35 ± 0.02 cdef |  | WIR453 | 0.22 ± 0.02 ab |  | WIR275 | 0.28 ± 0.02 bcde |  | WIR214 | 0.28 ± 0.00 a |  | WIR275 | 0.29 ± 0.02 bcdef |
| R | 0.54 ± 0.01 ab |  | WIR333 | 0.51 ± 0.04 cdef |  | ET-2 | -0.08 ± 0.01 ab |  | Ever 41 | -0.16 ± 0.01 efg |  | WIR276 | 0.33 ± 0.01 ab |  | WIR333 | 0.31 ± 0.01 def |  | WIR119 | 0.22 ± 0.00 ab |  | WIR333 | 0.25 ± 0.02 cde |  | WIR453 | 0.28 ± 0.02 a |  | WIR333 | 0.29 ± 0.02 bcdef |
| WIR119 | 0.54 ± 0.00 ab |  | WIR119 | 0.46 ± 0.02 def |  | G-1 | -0.09 ± 0.01 ab |  | PI468075 | -0.17 ± 0.01 efg |  | WIR333 | 0.33 ± 0.00 ab |  | WIR119 | 0.30 ± 0.01 def |  | R | 0.22 ± 0.00 ab |  | WIR119 | 0.22 ± 0.01 de |  | PI468075 | 0.28 ± 0.01 a |  | WIR119 | 0.24 ± 0.02 def |
| WIR276 | 0.54 ± 0.04 ab |  | WIR276 | 0.44 ± 0.09 def |  | WIR360 | -0.09 ± 0.01 ab |  | EF-1 | -0.17 ± 0.03 fg |  | WIR119 | 0.33 ± 0.00 ab |  | WIR276 | 0.29 ± 0.03 ef |  | WIR452 | 0.21 ± 0.02 ab |  | WIR276 | 0.21 ± 0.04 de |  | WIR119 | 0.28 ± 0.01 a |  | WIR452 | 0.24 ± 0.06 cdef |
| WIR452 | 0.51 ± 0.05 ab |  | WIR452 | 0.43 ± 0.07 ef |  | EF-1 | -0.09 ± 0.01 ab |  | EF-3 | -0.17 ± 0.01 g |  | WIR452 | 0.31 ± 0.02 ab |  | WIR452 | 0.29 ± 0.02 ef |  | WIR276 | 0.21 ± 0.02 ab |  | WIR452 | 0.21 ± 0.04 de |  | R | 0.28 ± 0.00 a |  | WIR276 | 0.23 ± 0.05 def |
| WIR214 | 0.51 ± 0.01 b |  | WIR274 | 0.38 ± 0.07 ef |  | Ever 71 | -0.09 ± 0.01 ab |  | ET-2 | -0.19 ± 0.02 g |  | WIR214 | 0.31 ± 0.00 b |  | WIR274 | 0.27 ± 0.03 f |  | WIR214 | 0.21 ± 0.00 ab |  | WIR274 | 0.19 ± 0.04 e |  | WIR276 | 0.27 ± 0.03 a |  | WIR274 | 0.21 ± 0.04 ef |
| WIR274 | 0.49 ± 0.03 b |  | WIR214 | 0.35 ± 0.02 f |  | EF-3 | -0.11 ± 0.01 b |  | Ever 71 | -0.19 ± 0.02 g |  | WIR274 | 0.30 ± 0.01 b |  | WIR214 | 0.26 ± 0.00 f |  | WIR274 | 0.20 ± 0.01 b |  | WIR214 | 0.17 ± 0.01 e |  | WIR274 | 0.27 ± 0.01 a |  | WIR214 | 0.18 ± 0.02 f |

^a^ Set 1 and Set 2 measured June 14 and August 31.

^b^ Ever 41 = Everglades 41, and Ever 71 = Everglades 71

^c^ Means of ± standard errors followed by different letters within columns are significantly different by Dunn test with Bonferroni adjustment. Non-parametric rank data were used for statistical analysis; however, untransformed data are presented.
